# DDAffinity: predicting the changes in binding affinity of multiple point mutations using protein 3D structure

**DOI:** 10.1093/bioinformatics/btae232

**Published:** 2024-06-28

**Authors:** Guanglei Yu, Qichang Zhao, Xuehua Bi, Jianxin Wang

**Affiliations:** School of Computer Science and Engineering, Central South University, Changsha 410083, China; Hunan Provincial Key Lab on Bioinformatics, Central South University, Changsha 410083, China; Medical Engineering and Technology College, Xinjiang Medical University, Urumqi 830017, China; School of Computer Science and Engineering, Central South University, Changsha 410083, China; Hunan Provincial Key Lab on Bioinformatics, Central South University, Changsha 410083, China; School of Computer Science and Engineering, Central South University, Changsha 410083, China; Hunan Provincial Key Lab on Bioinformatics, Central South University, Changsha 410083, China; Medical Engineering and Technology College, Xinjiang Medical University, Urumqi 830017, China; School of Computer Science and Engineering, Central South University, Changsha 410083, China; Hunan Provincial Key Lab on Bioinformatics, Central South University, Changsha 410083, China

## Abstract

**Motivation:**

Mutations are the crucial driving force for biological evolution as they can disrupt protein stability and protein–protein interactions which have notable impacts on protein structure, function, and expression. However, existing computational methods for protein mutation effects prediction are generally limited to single point mutations with global dependencies, and do not systematically take into account the local and global synergistic epistasis inherent in multiple point mutations.

**Results:**

To this end, we propose a novel spatial and sequential message passing neural network, named DDAffinity, to predict the changes in binding affinity caused by multiple point mutations based on protein 3D structures. Specifically, instead of being on the whole protein, we perform message passing on the *k*-nearest neighbor residue graphs to extract pocket features of the protein 3D structures. Furthermore, to learn global topological features, a two-step additive Gaussian noising strategy during training is applied to blur out local details of protein geometry. We evaluate DDAffinity on benchmark datasets and external validation datasets. Overall, the predictive performance of DDAffinity is significantly improved compared with state-of-the-art baselines on multiple point mutations, including end-to-end and pre-training based methods. The ablation studies indicate the reasonable design of all components of DDAffinity. In addition, applications in nonredundant blind testing, predicting mutation effects of SARS-CoV-2 RBD variants, and optimizing human antibody against SARS-CoV-2 illustrate the effectiveness of DDAffinity.

**Availability and implementation:**

DDAffinity is available at https://github.com/ak422/DDAffinity.

## 1 Introduction

Mutations, i.e. protein amino acid substitutions, play a crucial role in natural evolution by disrupting protein stability and protein–protein interactions (PPIs) as they can severely affect protein structure, function, expression, and solubility (Pires *et al.* 2014). A representative example is variants in receptor-binding domain (RBD) of the spike protein of severe acute respiratory syndrome coronavirus 2 (SARS-CoV-2), which affects its binding to the human angiotensin-converting enzyme 2 (ACE2) receptor and its recognition by human antibodies (Starr *et al.* 2022). Mutations in ACE2 are also used for engineering high-affinity therapeutic decoy receptor candidates (Chan *et al.* 2020). Therefore, understanding how mutations affect the changes in binding affinity is crucial for the development and optimization of a wide range of biotechnology products, including antibodies, industrial-grade enzymes, and other protein-based therapeutics and reagents (Rouet *et al.* 2014, Goldenzweig and Fleishman 2018).

Binding affinity, which spans more than nine orders of magnitude, is used to measure the binding strength between PPIs (Erijman *et al.* 2014). Precisely distinguishing the changes in binding affinity (i.e. ΔΔG) upon mutations is an important but still elusive and unsolved challenge in protein engineering. Much research has been devoted to developing computational tools, including traditional methods, machine learning and deep learning approaches.

Traditional methods, such as PoPMuSiC (Kwasigroch *et al.* 2002) and BeAtMuSiC (Dehouck *et al.* 2013), generally aim to understand the effects of single point mutations by comparing the statistical differences between wild-type and mutant. These traditional methods, also like Rosetta (Alford *et al.* 2017, Leman *et al.* 2020), FoldX (Delgado *et al.* 2019), etc., mainly use physical energy features to describe mutation effects, which often lead to increased computational costs associated with large-scale conformation space sampling and energy optimization.

Aside from traditional methods, machine learning approaches have been developed to predict protein mutation effects, which largely rely on feature engineering summarized as physicochemical, biophysical, and quantum mechanical characteristics of proteins. Two typical approaches for predicting the effects of single point mutations are mCSM (Pires *et al.* 2014) and INPS (Fariselli *et al.* 2015), where mCSM is based on graph-based features constructed from Cutoff Scanning Matrix (Pires *et al.* 2011), pharmacophore counts, and experimental conditions, while INPS constructs SVM regressions based on protein sequences. To represent the effects of multiple point mutations, mmCSM-PPI (Rodrigues *et al.* 2021) calculates the sum and average feature values of six categories, including dynamics, residue conservation, and residue environment properties, etc. Although widely used, the performance of machine learning methods heavily relies on expert knowledge, which limits their development with the rapid growth of available protein 3D structure data.

In recent years, the rapid accumulation of high resolution protein structures, aided by experimental methods such as X-ray crystallography, nuclear magnetic resonance (NMR), and cryo-electron microscopy (cryo-EM), has facilitated the widespread application of deep learning-based methods in the fields of protein mutation effects prediction. These approaches can be divided into end-to-end and pre-training based methods. End-to-end models typically incorporate wild-type and mutant protein structures as inputs. For example, DeepDDG (Cao *et al.* 2019) shares neural network parameters between each target-neighbor residue pair and considers the local environment of the mutant residues. Analysis indicates that the solvent accessible surface area (SASA) of the mutated residue, which determines the buried hydrophobic area, is a major determinant of protein stability. With mutant structures sampled by the Rosetta cartesian_ddg program (Park *et al.* 2016, Frenz *et al.* 2020), DDGPred (Shan *et al.* 2022) integrates inter-residue interaction features using geometric attention and physical energy terms to predict the changes in binding affinity with an anti-symmetric network. Taking sequences, structures, and energy features as input, UniBind (Wang *et al.* 2023) incorporates a dual-path neural network with attention mechanism, multi-task learning, and model ensemble to extract information for prediction of protein mutation effects from diverse biological datasets.

Pre-training based models for predicting protein mutation effects using protein 3D structures generally have the following two main objectives. First, it is how to construct an adaptive encoder to effectively represent the 3D structure of proteins. Second, it is how to design appropriate pretext tasks for large-scale unlabeled datasets (i.e. constructing efficient objective functions to supervise the pre-training process), which requires specialized architectures and large-scale training data, leading to increased computational costs associated with model design and implementation. GeoPPI (Liu *et al.* 2021) constructs a geometric encoder by incorporating graph attention network (GAT) to capture the structure features of protein complexes at atomic level. They optimize the conformation of amino acid sidechains through self-supervised learning and integrate gradient-boosting trees (GBT) to predict changes in binding affinity. The encoder of RDE-Network (Luo *et al.* 2023) first generates embeddings for each individual residue and each pair of residues with two multilayer perceptrons (MLPs), then utilizes a self-attention based network to transform these embeddings into hidden representations for prediction. DiffAffinity (Liu *et al.* 2023) uses a similar encoder network to RDE-Network to transform the structural context of mutation sites with a self-attention based network. The difference lies in the use of the Invariant Point Attention Module (IPA), which is part of the SE(3)-invariant network presented in AlphaFold2 (Jumper *et al.* 2021). In addition to above mentioned encoder networks, pre-training models typically estimate amino acid sidechain conformations to learn protein representations from large-scale unlabeled protein structures using different self-supervised learning techniques. Overall, these models focus on global dependencies modeling of PPIs and perform well on single point mutations.

However, the aforementioned approaches do not take into account the local and global synergistic epistasis of the covalent interactions as well as noncovalent interactions, which are inherent to the PPIs of multiple point mutations. To this end, we propose a novel distance-dependent and sequence-dependent message passing neural network (MPNN) incorporating biological domain knowledge to learn the PPIs systematically, named DDAffinity, to predict the changes in binding affinity caused by multiple point mutations using protein 3D structure, which is simple yet effective due to incorporating neighborhood message passing and aggregation on protein *k*-nearest residue graphs. Extensive experiments have been conducted to validate the superior performance of our method. Specifically, on the internal test set, compared with baseline studies on the prediction of multiple point mutation effects, DDAffinity achieves state-of-the-art performances on all evaluation metrics. On the external test set, DDAffinity demonstrates the best performance on the tasks of human antibody optimization, SARS-CoV-2 RBD variants mutation effects prediction, and multiple point mutation nonredundant blind testing as well.

In summary, our contributions are as follows. (i) we formulate protein multiple point mutation effects prediction as a structure representation learning problem on the *k*-nearest neighbor graph. Compared with existing methods, further dividing MPNN into spatial, sequential and long-range interactions for message passing and aggregation, and combining domain knowledge of PPIs can notably improve the prediction performance. (ii) We propose a novel two-step additive Gaussian noising strategy for the input and backbone atomic coordinates during training with the aim of data diversity and eliminating the inconsistency introduced during the energy optimization of mutant structure generation. (iii) We utilize residue centrality normalization to characterize residue burial, which has been shown significant effects on protein structure and stability. In conclusion, we have integrated domain knowledge that affects protein multiple point mutations into the design of our model, which demonstrates strong predictive performance and generalization on multiple metrics on both internal and external test set in a systematic way.

## 2 Materials and methods

### 2.1 Problem statement

Given the wild-type protein complex (WT) and mutant protein complex (MT), the changes in binding affinity upon mutations is calculated as follows:

(1) $$\Delta \Delta G=\Delta G_{\mathrm{mutant}}-\Delta G_{\mathrm{wild}-\mathrm{type}},$$

where $\Delta G_{\mathrm{wild}-\mathrm{type}}$ and $\Delta G_{\mathrm{mutant}}$ are the changes in Gibbs free energy of wild-type and mutant respectively. The sign of ΔΔG indicates whether the mutations are stable or not. Taking WT and MT as input, the global objective function of our approach is to apply a neural network **f** to approximate the solution of mutation effects prediction problem as follows:

(2) ΔΔG=f(WT,MT).

### 2.2 Datasets

The training and validation datasets utilized in this study are derived from SKEMPI2 (Jankauskaitė *et al.* 2019), which comprises 345 complexes and 7805 point mutations with experimentally determined ΔΔG values. SKEMPI2 is curated by calculating the average mutation effect for variants reported in multiple experiments, and its 3D structures have been deposited in the Protein Data Bank (PDB).

In addition to the overall SKEMPI2 dataset, two subsets of SKEMPI2 are used as benchmark datasets for comparing the predictive performance of DDAffinity with baseline models, namely S1131 and M1707. According to Xiong *et al.* (2017), S1131 is a subset with 1131 nonredundant interface single point mutations. M1707 is a subset with 1707 multiple point mutations, including 1337 multiple point mutations plus reverse mutations (Zhang *et al.* 2020). We also conduct experiment on a multiple point mutation nonredundant blind testing dataset at the mutation-level, which were proposed in mmCSM-PPI (Rodrigues *et al.* 2021) and also curated from SKEMPI2. We filter out problematic structures, and mark them as M1340 and M595. To estimate the anti-symmetric property, we use the Ssym dataset presented in Pucci *et al.* (2018), in which the proportion of direct and inverse variations is unbiased.

Furthermore, we also include two external validation datasets for case study. The first consists of 285 single point mutations of the ancestral Wuhan-Hu-1 RBD (PDB ID: 6M0J) (Starr *et al.* 2022), whose ΔΔG values have been experimentally quantified by deep mutational scanning techniques. The second contains 494 single point mutations of the SARS-CoV-2 RBD (PDB ID: 7FAE), which are ranked according to the predicted ΔΔG values, aiming to precisely identify the top five mutations that exhibit enhanced binding affinity.

In this study, mutant complex structures are generated by searching for rotamer library using BuildModel function of FoldX from wild-type complex crystal structures obtained from the PDB, and then are optimized using Optimize function of FoldX, which does a quick optimization to eliminate Van der Waals’ clashes by slightly moving all sidechains.

### 2.3 Model architecture

**Figure 1.**
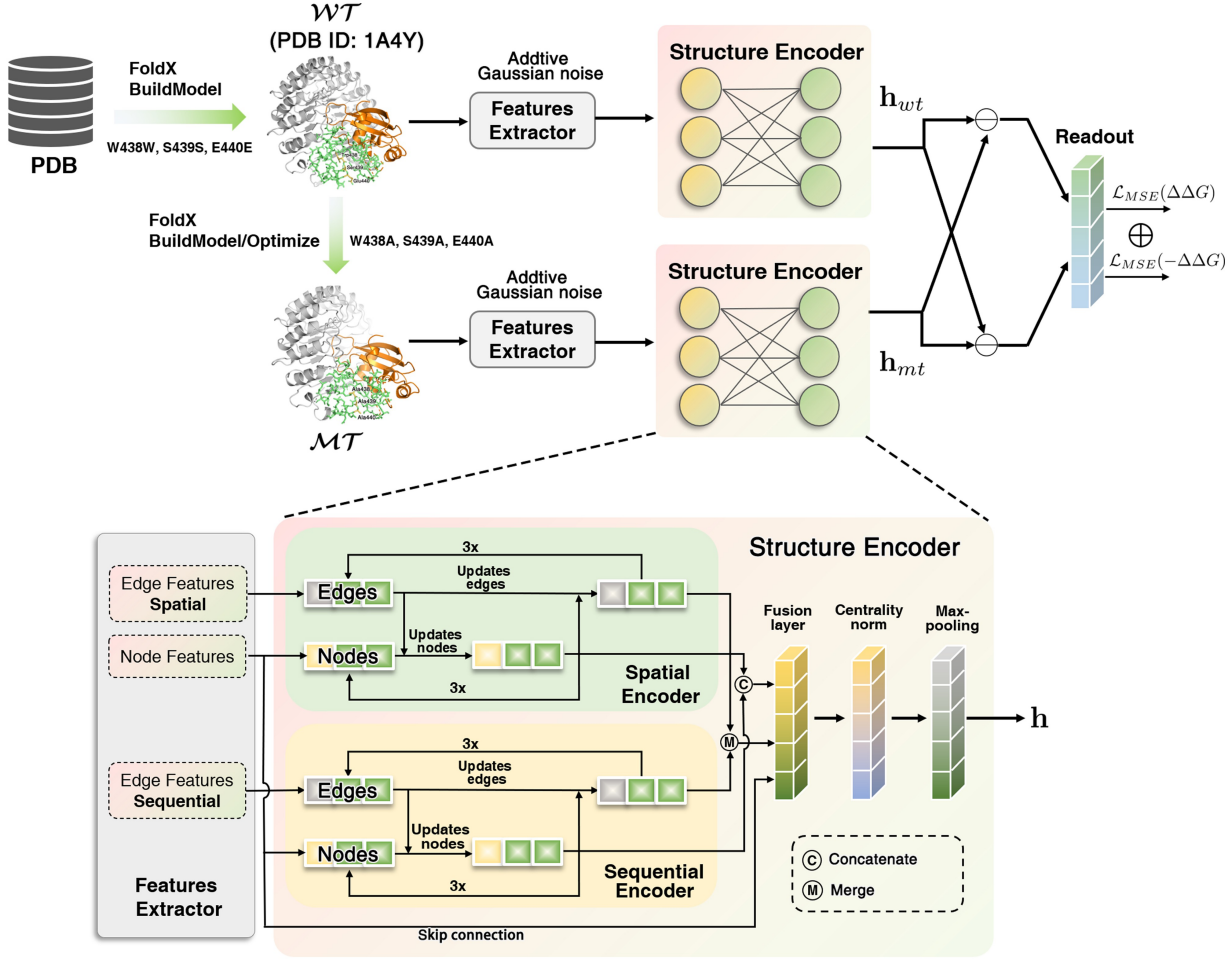
Overview of DDAffinity architecture. (a) Overall procedure of DDAffinity. (b) Detail components of structure encoder.

We combine sequential, spatial and physicochemical information into the *k*-nearest neighbor residue graph of proteins 3D structure for message passing and aggregation, which is novel and crucial for protein structure modeling, whereas previous works (Ingraham *et al.* 2019, Dauparas *et al.* 2022) only consider spatial message passing among residues or atoms. Figure 1 illustrates the composition of each module in DDAffinity. Given the *k*-nearest residues graph of wild-type and mutant protein complex, respectively, we now present how to design a sharing structure encoder in what follows.

#### 2.3.1 Structure encoder

##### 2.3.1.1 Protein structure graph

A protein complex is made up of multiple polypeptide chains linked together by noncovalent PPIs. We represent protein complex structure containing *L* residues as a *k*-nearest neighbors graph of spatially and sequentially aggregated components $G=(V\in R^{L\times d}$, $E:=\{E^{\mathrm{spatial}}$, $E^{\mathrm{sequential}}\})$ with node features $V=\{v_{1},\ldots ,v_{L}\}$ describing each residue and edge features $E={\left\{e_{ij}\right\}}_{j\in N(i,k)}$ capturing relationships between residue *i* and residue *j*, N(i,k) is the *k*-nearest neighbors of residue *i*. Here, the amino acid type of each residue *i* is in set {1,…,20}. $\chi_{i}\in [-\pi ,\pi )$ denotes the sidechain torsion angles of residue *i*. The number of $\chi_{i}$ ranges from 0 to 4 depending on its amino acid type. In this work, the Euclidean distance reflects the spatial relations between the β-carbon ($C_{\beta }$) coordinates of residues.

##### 2.3.1.2 Spatial and sequential encoder layer

The design of our proposed structure encoder architecture is inspired by the following two observations. Firstly, epitopes are commonly classified as linear epitopes, where antibodies bind to a contiguous stretch of residues in sequences through peptide bonds, and conformational epitopes, where antibodies bind to discontinuous residues in sequences but are in spatial proximity due to the folding of polypeptide chain (Zhang *et al.* 2012). Secondly, disulfide bonds are believed to decrease the conformational entropy and provide an increase in stability to the folded protein conformation (Flory 1956). However, the long-range interactions between protein partners, which have been shown to be the most discriminative features for protein binding affinity (Pires *et al.* 2014), are neglected in GNN models.

**Figure 2.**
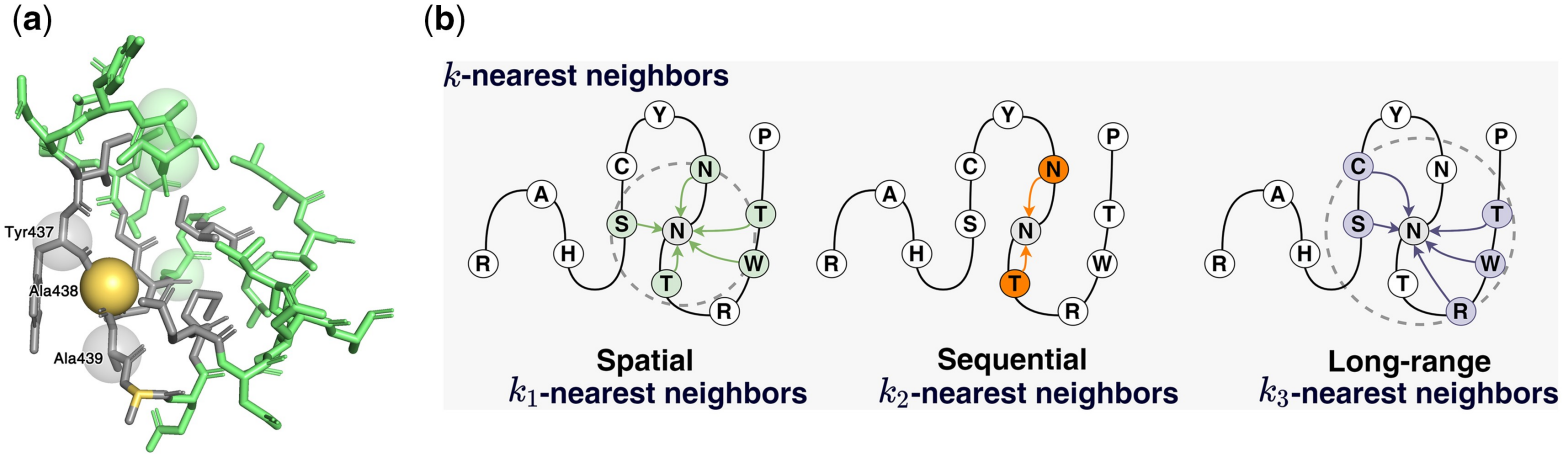
Illustration of *k*-nearest neighbors aggregation for spatial and sequential encoders. The $k_{1}$ (see (a) gray sticks and (b) left) and $k_{3}$-nearest residues (see (a) green balls and (b) right) for spatial encoder, and the $k_{2}$-nearest residues (see (a) gray balls and (b) center) for sequential encoder.

To thoroughly encode node features V and edge features E (see Section 2.3.2 for details of features extraction), we propose to adopt ProteinMPNN as the backbone of DDAffinity, which consists of a spatial encoder and a sequential encoder to aggregate V, E, and physicochemical attributes within the *k*-nearest neighbor graph derived from the protein 3D structures. Specifically, as shown in Fig. 2, we use three different neighbor residues to construct the *k*-nearest neighbor graph. (i) Spatial distance $k_{1}$. A residue will be connected to its $k_{1}$-nearest neighbors according to their spatial Euclidean distances. (ii) Sequential distance $k_{2}$. The linear interactions of residues are defined as the sequential distance between the residue $r_{i}$ and its sequence neighbors if their sequential distances are no more than $(k_{2}-1)/2$. (iii) Long-range distance $k_{3}$. For efficiently capturing those dependencies that are long-range in sequence but local in 3D Euclidean space, neighbors of residue $r_{i}$ are ranked in ascending order according to their Euclidean distances, and discarded if their sequence distances are not greater than $(k_{2}-1)/2$. After that, we select the $k_{3}$-nearest neighbors from the ordered neighbor list. In summary, $k=k_{1}+k_{2}+k_{3}$.

Since we utilize weight sharing between the structure encoders of WT and MT, the following takes the structure encoder of WT as an example. Different from ProteinMPNN, Pre-Norm (applied to the input) (Domhan 2018, Wang *et al.* 2021) and ReZero (applied to residual architectures) (Bachlechner *et al.* 2021) training strategies are considered to improve training efficiency and convergence speed. Denote $h\_V_{i}^{\left(l\right)}$ and $h\_E_{ij}^{\left(l\right)}$ as the input to the (l+1)th block and define $h\_V_{i}^{\left(0\right)}=v_{i}$ and $h\_E_{ij}^{\left(0\right)}=e_{ij}$. The (l+1)th block for node updates works as follows,

(3) $$m_{ij}^{\left(l\right)}=\mathrm{MLP}(\mathrm{Cat}(LN(h\_V_{i}^{\left(l\right)}),LN(h\_V_{j}^{\left(l\right)}),h\_E_{ij}^{\left(l\right)})),$$

(4) $$\begin{array}{c} h\_V_{i}=h\_V_{i}^{\left(l\right)}+\alpha^{\left(l\right)}d\mathrm{ropout}(\frac{\sum_{j\in N(i,k)}m_{ij}^{\left(l\right)}}{\lambda }), \end{array}$$

(5) $$\begin{array}{c} h\_V_{i}^{\left(l+1\right)}=h\_V_{i}+\alpha^{\left(l\right)}d\mathrm{ropout}(\mathrm{FFN}(LN(h\_V_{i}))), \end{array}$$

where MLP(·) is multilayer perceptron of three layers, Cat(·) denotes the concatenation operation, LN(·) is layer normalization, FFN(·) is feed-forward network, $\alpha^{\left(l\right)}$ is learnable scalar, and λ=30 is a scaling constant.

For input $h\_V_{i}^{\left(l+1\right)}$ and $h\_E_{ij}^{\left(l\right)}$, the (l+1)th block for edge updates works as follows,

(6) $$m_{ij}^{\left(l\right)}=\mathrm{MLP}(\mathrm{Cat}(h\_V_{i}^{\left(l+1\right)},h\_V_{j}^{\left(l+1\right)},LN(h\_E_{ij}^{\left(l\right)}))),$$

(7) $$\begin{array}{c} h\_E_{ij}^{\left(l+1\right)}=h\_E_{ij}^{\left(l\right)}+\mathrm{dropout}(m_{ij}^{\left(l\right)}). \end{array}$$

Then we obtain edge hidden representation *h_E* and node hidden representation *h_V* from the last block output of the structure encoder.

##### 2.3.1.3 Fusion layer

To prevent gradients vanishing when network going deeper or using max-pooling operation, we add skip connection between the input and output of structure encoder to avoid degradation as shown in Fig. 1. For input $h\_V^{\left(0\right)}$, $h\_V^{\mathrm{spatial}}$, $h\_V^{\mathrm{sequential}}$, $h\_E^{\mathrm{spatial}}$, and $h\_E^{\mathrm{sequential}}$, the fusion operation works as follows,

(8) $$h\_V_{i}^{\mathrm{enc}}=\mathrm{Cat}(h\_V_{i}^{\mathrm{spatial}},h\_V_{i}^{\mathrm{sequential}})W^{2d\times d},$$

(9) $$\begin{array}{c} h\_E_{ij}^{\mathrm{enc}}=\mathrm{Merge}(h\_E_{ij_{1}}^{\mathrm{spatial}},h\_E_{ij_{2}}^{\mathrm{sequential}}), \end{array}$$

(10) $$\begin{array}{c} m_{ij}=\mathrm{MLP}(\mathrm{Cat}(h\_V_{i}^{\mathrm{enc}},h\_V_{j}^{\left(0\right)},h\_V_{j}^{\mathrm{enc}},h\_E_{ij}^{\mathrm{enc}})), \end{array}$$

(11) $$\begin{array}{c} h\_V_{i}=LN(h\_V_{i}^{\mathrm{enc}}+\mathrm{dropout}(\sum_{j\in N(i,k)}m_{ij})), \end{array}$$

where Merge(·) represents the merging operation on spatial and sequential edge representations, i.e. $h\_E_{ij}^{\mathrm{enc}}\in R^{L\times k\times d}$, $h\_E_{ij_{1}}^{\mathrm{spatial}}\in R^{L\times (k_{1}+k_{3})\times d}$, $h\_E_{ij_{2}}^{\mathrm{sequential}}\in R^{L\times k_{2}\times d}$.

##### 2.3.1.4 Residue centrality norm layer

Analysis showed that residue burial has significant effects on protein structure and stability (Nisthal *et al.* 2019). In this study, we propose normalized residue centrality to measure the different contributions among residues to ΔΔG. Residue centrality is defined as the number of $C_{\beta }$ atoms within a 10 Å distance ball of the query residue’s $C_{\beta }$ atom, exclude itself (McPartlon and Xu 2023). For the residue of missing $C_{\beta }$ atom, coordinates of $C_{\beta }$ are calculated using virtual $C_{\beta }$ coordinates [see Dauparas *et al.* (2022) for details].

To quantify residue burial, we propose residue centrality normalization to regular the binding affinity difference of each residue. The normalized residue centrality represents its *k*-nearest neighbor weight in the protein complex, which is defined as,

(12) $${\hat{c}}_{i}=\frac{\sum_{j\in N(i,k)}\left(c_{j}+w_{j}\right)}{\max_{t\in \{L\}}\sum_{j\in N(t,k)}\left(c_{j}+w_{j}\right)},$$

where ${\hat{c}}_{i}$ is max-norm regularization of residue $r_{i}$, $c_{j}$ is residue centrality of residue $r_{j}$, $w_{j}$ is learnable scalar indexed by the *k*-nearest neighbors, N(i,k) is the *k*-nearest neighbors of residue $r_{i}$ which can be seen as “residue local environment”, i∈{L}≜{1,2,…,L}, *L* is the length of clipped residue patch. Formally, the residue centrality norm layer used in our model is defined as,

(13) $$\tilde{V}=\hat{R}(h\_V),$$

where $\hat{R}(\cdot )$ denotes residue centrality normalization operation, and $\tilde{V}$ is the final node representation.

##### 2.3.1.5 Readout layer

After obtaining the hidden embeddings of residues, the structure encoder applies max-pooling to the node hidden embeddings to obtain a global protein representations. By sharing the same structure encoder architecture between the wild-type complex structure WT and the mutant complex structure MT, we obtain the representations of the wild-type structure and mutant structure, respectively.

Then we subtract the representation of the wild-type structure from the mutant representation and feed it into another MLP to predict ΔΔG. To enforce anti-symmetry, we exchange the wild-type and mutant to predict  − ΔΔG, and compute (ΔΔG − ( − ΔΔG))/2 as the final prediction. The network is trained using the MSE loss.

#### 2.3.2 Edge features and node features

##### 2.3.2.1 Residue-pairwise edge features

As for residue $r_{i}$ and its *k*-nearest neighbor residues $r_{j}$, the edge feature $e_{ij}$ is defined over the local coordinate frame $O_{i}=[b_{i}n_{i}b_{i}\times n_{i}]$ at residue $r_{i}$ [details in Ingraham *et al.* (2019)]. First, we decompose $e_{ij}$ into five components: distance, direction, orientation, amino acid-pair, and position as,

(14) $$\begin{array}{c} e_{ij}=\mathrm{Cat}(e_{ij}^{\mathrm{distance}},e_{ij}^{\mathrm{direction}},e_{ij}^{\mathrm{orientation}},\\ e_{ij}^{aa\_\mathrm{pair}},e_{ij}^{\mathrm{position}}). \end{array}$$

Then, the edge embedding $h\_E_{ij}$ is obtained according to $h\_E_{ij}=\text{GELU}\left(e_{ij}\;W^{1}\right)W^{2}\in R^{k\times d}$, where *L* is the patch size, *k* is the number of nearest neighbors, and *d* is the dimension of hidden embedding. And last, we derive each component of $e_{ij}$ from $O_{i}$ to $O_{j}$ as follows.

Firstly, we encode the Euclidean distance using Gaussian Basis Kernel function (Gao *et al.* 2023) to reflect the spatial relation between any atom-pair in the backbone atoms set $A=\{N,C_{\alpha },C,O,C_{\beta }\}$ as follows,

(15) $$e_{ij}^{\mathrm{distance}}=\psi \left(\left||x_{j}-x_{i}|\right|\right).$$

The detailed steps are as follows,

(16) $$||X_{j}-X_{i}||=\gamma_{\left(a_{i},a_{j}\right)}||x_{j}-x_{i}||+\beta_{\left(a_{i},a_{j}\right)},$$

(17) $$\begin{array}{c} \psi_{\left(a_{i},a_{j}\right)}^{k}=-\frac{1}{\sqrt{2\pi }|\sigma^{k}|}\text{exp}\;\left(-\frac{1}{2}{\left(\frac{||X_{j}-X_{i}||-\mu^{k}}{\left|\sigma^{k}\right|}\right)}^{2}\right), \end{array}$$

(18) $$\begin{array}{c} \psi_{\left(a_{i},a_{j}\right)}={\left[\psi_{\left(a_{i},a_{j}\right)}^{1};\ldots ;\psi_{\left(a_{i},a_{j}\right)}^{K}\right]}^{⊤}, \end{array}$$

(19) $$\begin{array}{c} \psi \left(\left||x_{j}-x_{i}|\right|\right)=\mathrm{Cat}(\{\psi_{\left(a_{i},a_{j}\right)}|i,j=1,2,\ldots ,5\}), \end{array}$$

where $x_{i},x_{j}$ are coordinates of atoms $a_{i},a_{j}\in A$ respectively, *K* is the number of Gaussian Basis kernels, $\gamma_{\left(a_{i},a_{j}\right)},\beta_{\left(a_{i},a_{j}\right)}$ are learnable scalars indexed by the $\left(a_{i},a_{j}\right)$, and $\mu^{k},\sigma^{k}$ are learnable kernel center and learnable scaling factor of the *k*th Gaussian Basis Kernel.

Secondly, the details of $e_{ij}^{\mathrm{direction}}$ and $e_{ij}^{\mathrm{orientation}}$ are described in Ingraham *et al.* (2019), and are briefly described as:

(20) $$e_{ij}^{\mathrm{direction}}=O_{i}^{T}\frac{x_{j}-x_{i}}{\left||x_{j}-x_{i}|\right|},$$

(21) $$\begin{array}{c} e_{ij}^{\mathrm{orientation}}=q\;(O_{i}^{T}O_{j}), \end{array}$$

where $O_{i}$ defines a local coordinate system at residue $r_{i}$, $e_{ij}^{\mathrm{direction}}$ represents the relative direction of $x_{j}$ in the reference frame of $\left(x_{i},O_{i}\right)$, and q(·) defines the orientation with quaternion representation of spatial rotation matrix $O_{i}^{T}O_{j}$.

Thirdly, we map the residue-pair interactions for certain residue and its *k*-nearest neighbors to $\tau_{\left(r_{i},r_{j}\right)}$ as follows,

(22) $$\tau_{\left(r_{i},r_{j}\right)}=\mathrm{Merge}(\{r_{i}*21+r_{j}|j\in N(i,k)\}),$$

where $r_{i},r_{j}\in \{0,1,\ldots ,20\}$ with one additional dimension for unknown residue type, and $\tau_{\left(r_{i},r_{j}\right)}\in {\left\{0,1,\ldots ,440\right\}}^{k}$, then we have residue-pair embeddings $e_{ij}^{aa-\mathrm{pair}}\in R^{L\times k\times d}$ as follows,

(23) $$e_{ij}^{aa-\mathrm{pair}}=f^{aa-\mathrm{pair}}(\tau_{\left(r_{i},r_{j}\right)}).$$

Finally, unlike the method of masking interchain residues in Dauparas *et al.* (2022), we utilize an additional binary embedding to indicate whether the interacting residue-pairs are interchain or intrachain residues. And when encoding residue positions, we maintain the relative positions of residues within intrachain residues capped at ±32. Thus, the positions of residues are encoded as,

(24) $$e_{ij}^{\mathrm{position}}=f^{\mathrm{relative}}(p_{r_{j}}-p_{r_{i}})+f^{\mathrm{chains}}(c_{\left(r_{i},r_{j}\right)}),$$

where $p_{r_{i}}$ is sequence position of residue $r_{i}$, $|p_{r_{j}}-p_{r_{i}}|\leq 32$. $c_{\left(r_{i},r_{j}\right)}=1$ means residue $r_{i}$ and $r_{j}$ are in the same chain, otherwise $c_{\left(r_{i},r_{j}\right)}=0$.

##### 2.3.2.2 Residue node features

To generate node features, we calculate the backbone dihedral angles $\left(\phi_{i},\psi_{i}\right)$ and sidechain dihedral angles $\left(\chi_{i}^{\left(1\right)},\chi_{i}^{\left(2\right)},\chi_{i}^{\left(3\right)},\chi_{i}^{\left(4\right)}\right)$ of residue $r_{i}$. Subsequently, we embed these angles onto the torus space, denoted as $\{\;\text{sin}\;,\;\text{cos}\;\}\times (\phi_{i},\psi_{i},\chi_{i}^{\left(1\right)}$, $\chi_{i}^{\left(2\right)},\chi_{i}^{\left(3\right)},\chi_{i}^{\left(4\right)})$.

Furthermore, we follow the approach outlined in Jin *et al.* (2022) to construct the physicochemical features of residues, which consist of six features: binary polarity ∈{0,1}, binary hydrogen bond donor ∈{0,1}, binary hydrogen bond acceptor ∈{0,1}, charge ∈{ − 1,0,1}, and hydropathy ∈[ − 4.5,4.5] and volume ∈[60.1,227.8] encoded by another Gaussian radial basis function.

## 3 Results

In this section, we present our experimental setup for training (Section 3.1) and evaluation metrics (Section 3.2) on two tasks: ΔΔG prediction and human antibody optimization. In predicting ΔΔG, we leverage the overall SKEMPI2, S1131, M1707, and M1340 for internal validation, and M595 for nonredundant blind testing (Section 3.3). In our case study, we focus on predicting the mutation effects of SARS-CoV-2 RBD and optimizing human antibody against SARS-CoV-2 tasks (Section 3.4). For anti-symmetric property validation, the results are listed in Supplementary Note S5. In addition, we perform internal experiments to gain better insights of our model (Sections 3.5 and 3.6).

### 3.1 Experimental setup

To fairly evaluate the performance of DDAffinity, we followed the same strategy used in RDE-Network splitting the training and testing sets. Specifically, we used a ten-fold cross-validation to train the model and assessed the performance based on complex-level without protein structure overlapping. Therefore, ten models were trained.

In our experiments, we implemented a three-layer structure encoder with hidden dimension of 128. The *k*-nearest parameters were set to $k_{1}=16$, $k_{2}=3$, and $k_{3}=7$. The number of Gaussian Basis kernels was set to 16. The training process consisted of up to 100,000 iterations (55 epochs) using the Adam optimizer with setting $\beta_{1}=0.9$, $\beta_{2}=0.999$, $\epsilon =1\times 10^{-8}$, and a learning rate of $6\times 10^{-4}$. In this work, we used an early-stopping strategy to accelerate the optimization process, that was, to halt the optimization process when the test score of the internal cross-validation for model selection stops improving (patience of 10 validation steps). Validation steps were performed every 1000 training steps to monitor the optimization procedure, and the batch size was set to 32 unless otherwise noted. Dropout ratios for the input embeddings, encoder layers, and fusion layer were set to 0.0, 0.1, and 0.1, respectively. Our models were implemented using PyTorch deep learning framework, and all experiments were running on a single A100 GPU.

When given WT and MT, we selected mutant residues as anchors and clipped WT and MT into residue patches, which are the 256 nearest neighbors of the mutant residues based on $C_{\beta }$ distances of inter-residues respectively, including the mutant residues. To improve the performance and generalization of DDAffinity, we implemented a two-step additive Gaussian noising strategy for the atomic coordinates of residues. Firstly, the crystallization of wild-type protein is generally not a physiological or an energy-optimal state. To reduce the inconsistencies introduced during the energy optimization process for generating mutant structures, as well as the inherent differences in proteins, the additive Gaussian noise (std=0.2Å) was combined with all input atomic coordinates, which yields the perturbed backbone dihedrals (ϕ,ψ) and sidechain dihedrals $\left(\chi^{\left(1\right)},\chi^{\left(2\right)},\chi^{\left(3\right)},\chi^{\left(4\right)}\right)$. Secondly, inspired by the ideas of ProteinMPNN that can improve predictive performance and make prediction algorithm more robust, we also incorporated Gaussian noise (std=0.2Å) to the atomic coordinates of protein backbone atom set $A=\{N,C_{\alpha },C,O,C_{\beta }\}$. Importantly, this perturbation was conducted without updating the backbone dihedrals and sidechain dihedrals. Additionally, we only implemented above mentioned two-step additive Gaussian noising strategy during training.

### 3.2 Evaluation metrics

In this paper, five evaluation metrics, including Pearson’s correlation coefficient (r), Spearman’s rank correlation coefficient (ρ), Root Mean Square Error (RMSE), Mean Absolute Error (MAE), Area Under the Receiver Operating Characteristic Curve (AUROC) were used in ΔΔG prediction task, with r as the primary metric. For evaluating the optimization of human antibody task, we utilized Hits@k (Ali *et al.* 2022) which denotes the ratio of the favorable mutations set $K_{\mathrm{favorable}}$ that have been ranked among the top-k test dataset, i.e.

(25) $$\text{Hits}@k=\frac{\left|\{t\in K_{\mathrm{favorable}}\;|\;\mathrm{rank}(t)\leq k\}\right|}{\left|K_{\mathrm{favorable}}\right|}.$$

When calculating AUROC, mutations were classified according to the sign of ΔΔG, i.e. whether mutations are stable or not. To measure the anti-symmetry, we calculated r and bias (〈δ〉) between the predicted ΔΔG of the direct ($\Delta \Delta G^{\mathrm{dir}}$) and inverse ($\Delta \Delta G^{\mathrm{inv}}$) variations as mentioned in Montanucci *et al.* (2019). And the 〈δ〉 is defined as,

(26) $$\langle \delta \rangle =\frac{\sum_{i=1}^{N}(\Delta \Delta G_{i}^{\mathrm{dir}}-(-\Delta \Delta G_{i}^{\mathrm{inv}}))}{2N}.$$

### 3.3 Prediction of the changes in binding affinity

To evaluate the performance of DDAffinity, we conducted comparison experiments using 3-fold, 5-fold, and 10-fold cross-validation on SKEMPI2 with eight baselines in four categories. DDAffinity achieves state-of-the-art performances in all evaluation metrics on the multiple point mutation subset. In 3-fold cross-validation, DDAffinity outperforms other methods across all metrics for the ΔΔG prediction on the single point mutation, multiple point mutation, and overall subset of SKEMPI2. With respect to the main evaluation metric of r, it is 0.624, 0.661, and 0.647, respectively, which are significant higher than baseline methods. In 5-fold cross-validation, DDAffinity also demonstrates better performance than baseline on multiple point mutation and overall subsets, with r values of 0.654 and 0.658, respectively, higher than DDGPred. In 10-fold cross-validation, DDAffinity achieves r of 0.693, ρ of 0.640, RMSE of 1.944, MAE of 1.496, and AUROC of 0.812, respectively, surpassing the pre-trained approaches on the multiple point subset. In summary, DDAffinity exhibits strong generalization performance, particularly demonstrating a significant performance advantage for the multiple point mutation prediction. The results are shown in Table 1 and visualized in Supplementary Note S4.

**Table 1.** Evaluation on the SKEMPI2 dataset.^a^

Category	Method	Fold	Mutations	r ↑	ρ ↑	RMSE↓	MAE↓	AUROC↑	Reference
Energy function	FoldX^b^	–	Overall	0.319	0.416	1.959	1.357	0.671	Delgado *et al.* (2019)
			Single	0.315	0.361	1.651	1.146	0.657	
			Multiple	0.256	0.418	2.608	1.926	0.704	
	Rosetta^b^		Overall	0.311	0.346	1.617	1.131	0.656	Alford *et al.* (2017)
			Single	0.325	0.367	1.183	0.987	0.674	
			Multiple	0.199	0.23	2.658	2.024	0.621	
	flex ddG^b^		Overall	0.402	0.427	1.587	1.102	0.675	Barlow *et al.* (2018)
			Single	0.425	0.431	1.457	0.997	0.677	
			Multiple	0.398	0.419	1.765	1.326	0.669	
Sequence based	ESM-1v^b^	3	Overall	0.192	0.157	1.961	1.368	0.541	Meier *et al.* (2021)
			Single	0.191	0.157	1.723	1.192	0.549	
			Multiple	0.192	0.175	2.759	2.119	0.542	
	ESM-IF^b^		Overall	0.319	0.281	1.886	1.286	0.590	Hsu *et al.* (2022)
			Single	0.296	0.287	1.673	1.137	0.605	
			Multiple	0.326	0.335	2.645	1.956	0.637	
End-to-end	DDAffinity		Overall	**0.647**	**0.507**	**1.576**	**1.151**	**0.724**	
			Single	**0.624**	**0.436**	**1.359**	**0.993**	**0.693**	
			Multiple	**0.661**	**0.602**	**2.025**	**1.540**	**0.791**	
End-to-end	DDGPred^b^	5	Overall	0.630	0.400	**1.313**	**0.995**	0.696	Shan *et al.* (2022)
			Single	**0.652**	0.359	**1.309**	**0.936**	0.656	
			Multiple	0.591	0.503	2.181	1.670	0.759	
	DDAffinity		Overall	**0.658**	**0.522**	1.557	1.146	**0.732**	
			Single	0.644	**0.480**	1.331	0.979	**0.717**	
			Multiple	**0.654**	**0.568**	**2.040**	**1.574**	**0.769**	
Pre-trained	RDE-Network^c^	10	Overall	0.639	0.554	1.590	1.125	0.742	Luo *et al.* (2023)
			Single	0.648	**0.520**	1.324	0.946	**0.731**	
			Multiple	0.599	0.572	2.161	1.612	0.774	
	DiffAffinity^c^		Overall	0.669	**0.555**	1.537	**1.110**	0.744	Liu *et al.* (2023)
			Single	**0.656**	0.517	**1.312**	**0.944**	0.726	
			Multiple	0.671	0.597	2.001	1.540	0.796	
End-to-end	DDAffinity		Overall	**0.681**	0.553	**1.513**	1.113	**0.745**	
			Single	0.654	0.492	1.315	0.967	0.716	
			Multiple	**0.693**	**0.640**	**1.944**	**1.496**	**0.812**	

a Bold values indicate the best results. The dash sign indicates that cross-validation is not applicable for the energy function methods.

b Results are from Liu *et al.* (2023).

c Results are from released source code.

Subsequently, we performed complex-level validation on subsets S1131 and M1707 to compare the predictive performance of DDAffinity with eight published models which were introduced in Wang *et al.* (2023) (Table 2). Our results demonstrate that DDAffinity is able to make predictions with notably higher predictive performance than other recently proposed methods for protein multiple point mutation effects prediction, as well as for the prediction of protein single point mutation effects.

**Table 2.** Comparison of r at complex-level for S1131 and M1707.^a^

Category	Method	r↑		Reference
		S1131	M1707	
Energy function	FoldX^b^	0.46	0.49	Delgado *et al.* (2019)
	Rosetta^b^	0.36	–	Leman *et al.* (2020)
Pre-trained	GeoPPI^b^	0.58	0.74	Liu *et al.* (2021)
	RDE-Network^c^	0.72	0.72	Luo *et al.* (2023)
	DiffAffinity^c^	0.76	0.72	Liu *et al.* (2023)
End-to-end	TopGBT^b^	0.32	–	Wang *et al.* (2020)
	UniBind^b^	0.69	0.78	Wang *et al.* (2023)
	DDGPred^b^	0.65	0.59	Shan *et al.* (2022)
	Ours	**0.80**	**0.80**	

a Bold values indicate the best results. The dash sign indicates the results of the corresponding methods are not available.

b Results are from Wang *et al.* (2023).

c Results are from released source code.

The performance of DDAffinity was further evaluated by performing training on the multiple point mutation dataset M1340 and nonredundant blind testing on M595 at mutation-level, which were two subsets derived from SKEMPI2. DDAffinity also exhibits notable improved predictive performance compared to baselines (Table 3).

**Table 3.** Performance comparison of nonredundant blind testing on the multiple point mutation dataset M595.

Method	r ↑	ρ ↑	RMSE↓	AUROC↑
FoldX^a^	0.39	0.37	5.27	0.22
Discovery studio^a^	0.39	0.41	3.07	0.66
mmCSM-PPI^a^	0.70	0.64	2.02	0.72
Ours	**0.74**	**0.70**	**1.91**	**0.83**

a Results are from Rodrigues *et al.* (2021).

Here, we propose a dataset partitioning scheme to minimize overlapping proteins between the testing and training sets, and we compare DDAffinity with the energy-function method FoldX on this dataset. The results are shown in Supplementary Table S1, and indicate that our model outperforms FoldX under the condition of reduced protein fold overlap (see Supplementary Note S1 for more details).

In addition, considering the use of ProteinMPNN as the backbone of DDAffinity, we have compared the performance of DDAffinity with ProteinMPNN. Taking the wild-type structure and the mutant structure as inputs, ProteinMPNN can obtain the score of the wild-type and the mutant, respectively, and subtracting the score of the mutant structure from the score of the wild-type structure gives the change in the score of the mutation. The comparison results on the SKEMPI2 dataset are shown in Supplementary Note S2. The results show that DDAffinity gives better predictions than ProteinMPNN.

### 3.4 Case study

To interpretate the evolution of SARS-CoV-2 and epistasis phenomenon, Starr *et al.* (2022) performed deep mutational scanning to experimentally quantify the effects of site-saturation mutagenesis on ACE2 binding to the ancestral Wuhan-Hu-1 RBD (PDB ID: 6M0J). According to Wang *et al.* (2023), 15 crucial mutation sites have been identified, which result in 285 point-mutations in total. DDAffinity exhibits superior performance with r=0.456, outperforming all baseline models (Table 4).

**Table 4.** Pearson’s correlation coefficient of mutation effects on binding affinity of SARS-CoV-2 RBD.

Method	FoldX^a^	RDE-Network^b^	DiffAffinity^b^	Ours
r ↑	0.385	0.403	0.305	**0.456**

a Results are from Liu *et al.* (2023).

b Results are from released source code.

In DDGPred (Shan *et al.* 2022), the authors reported five favorable point-mutations (PDB ID: 7FAE, including TH31W in CDR1, AH53F and NH57L in CDR2, RH103M and LH104F in CDR3) in the complementarity determining regions (CDRs) of the human antibody P36-5D2 against SARS-CoV-2. Thus, the task is to search out these favorable mutations within all 494 mutations of the CDRs. Specifically, these mutations are ranked based on predicted ΔΔG values of DDAffinity, with the most favorable (lowest ΔΔG values) mutation located at the top. As shown in Table 5, DDAffinity successfully identifies three of five favorable mutations, which obtains a best performance of 0.6 Hits@50 score. Specifically, DDAffinity ranks all five favorable mutations in the top 50%, which consistently outperforms all the baseline approaches and indicates its robustness and effectiveness in optimizing human antibodies task.

**Table 5.** Rankings of five favorable mutations in the CDRs of human antibody P36-5D2 against SARS-CoV-2 RBD.

Method	TH31W	AH53F	NH57L	RH103M	LH104F	Hits@50↑
FoldX^a^	4.25%	14.57%	2.43%	27.13%	63.77%	0.4
RDE-network^a^	5.06%	12.15%	55.47%	50.61%	9.51%	0.4
DiffAffinity*^a^	7.29%	0.81%	19.03%	84.21%	28.54%	0.4
DiffAffinity^a^	7.28%	3.64%	18.82%	81.78%	10.93%	0.4
Ours	41.90%	1.62%	13.36%	6.68%	6.88%	**0.6**

a Results are from Liu *et al.* (2023).

### 3.5 Ablation study

To demonstrate the added value of individual modules of DDAffinity on multiple point mutations, we conducted the following ablation experiments. We assessed the effects of five parts of the DDAffinity architecture as follows: residue centrality normalization (denoted as RC.), sequential interaction module (denoted as SI.), long-range interaction module (denoted as LR.), additive Gaussian noising strategy for input atomic coordinates (denoted as IA.), additive Gaussian noising strategy for backbone atomic coordinates (denoted as BA.), and the edge features of direction and orientation in local coordinate frame (denoted as DO.). The results are shown in Table 6.

**Table 6.** Ablation study on multiple point mutation subset of SKEMPI2.

Method	r ↑	ρ ↑	RMSE↓	MAE↓	AUROC↑
DDAffinity	**0.683**	**0.619**	**1.964**	**1.521**	**0.805**
w/o RC.	0.675	0.606	1.984	1.532	0.797
w/o SI.	0.646	0.569	2.078	1.617	0.781
w/o LR.	0.654	0.584	2.052	1.598	0.790
w/o IA.	0.637	0.548	2.113	1.636	0.760
w/o BA.	0.640	0.563	2.107	1.625	0.777
w/o DO.	0.682	0.599	1.974	1.539	0.797

From it, we have the following observations: (i) removing each module will lead to varying degrees of performance degradation, which indicates the positive effect of above components. (ii) For DDAffinity without IA. and BA., we observe a significant decrease in correlation and notable increase in error performance, which demonstrates that the two-step additive Gaussian noising strategy provides powerful generalization ability and robustness due to the ability to focus more on overall topological features rather than fine local structural details. (iii) DDAffinity without RC. performs slightly worse than DDAffinity, indicating the necessity of quantitative residue burial using residue centrality normalization to balance different contributions of residues. (iv) Due to the lack of local interactions in sequence and global interactions in long-range, we also see a performance decline for DDAffinity without SI. and LR. (v) The decrease after removing DO. suggests that edge features of direction and orientation do give a performance boost to the model. In summary, each module demonstrates the reasonable design of our method.

### 3.6 Exploration of the effect of *k* on model performance

The *k* is a critical parameter in the construction of *k*-nearest neighbor residue graphs. In this study, we set $k=k_{1}+k_{2}+k_{3}$, where $k_{1},k_{2}$, and $k_{3}$ represents the number of spatial nearest residues, sequential nearest residues, and long-range nearest residues, respectively.

For all subsets of SKEMPI2, we found that the best performance is achieved when $k_{1}=16$, $k_{2}=3$, and $k_{3}=7$, except for $k_{1}=24$ and $k_{3}=9$ on multiple point mutation subset (see Supplementary Note S3 for more details). To balance between the performance and efficiency, we set $k_{1}=16$, $k_{2}=3$, and $k_{3}=7$ as the default hyperparameters setting of our model when comparing with baselines in Section 3.3. By comparing the prediction results of single point mutations with multiple point mutations, we observed that multiple point mutations tend to aggregate residues from more distant than single point mutations. We attribute this to the co-evolutionary constraints of multiple point mutations, which may result in more stable protein 3D structures than single point mutations, as described in Section 3.3.

## 4 Conclusion

In this paper, we propose DDAffinity based on *k*-nearest neighbor residue graph, which aggregates information from spatial and sequential MPNN for accurately predicting changes in binding affinity caused by multiple point mutations. Compared with the benchmark studies, DDAffinity achieves high correlation and low error performance on the ΔΔG prediction task, which demonstrates the benefits of the proposed model architecture in correctly capturing the local and global synergistic epistasis property of PPIs in terms of covalent interactions and noncovalent interactions inherent in multiple point mutations. Furthermore, DDAffinity could be well applied for the mutation effects prediction of SARS-CoV-2 RBD variants and the optimization of human antibody against SARS-CoV-2.

Experimental results demonstrate that the large improvement margin is due to incorporate of additive Gaussian noising strategy during training. Ablation study shows that removing each part of DDAffinity will lead to a performance degradation on all performance metrics, which proves the positive effect of our proposed components. In addition, we propose residue centrality normalization as a quantitative substitute for residue burial to measure the different contributions of residues to ΔΔG. In summary, this article offers a novel protein structure encoder framework based on spatial and sequential MPNN, combined with residue centrality normalization and two-step additive Gaussian noising strategy to predict the changes in binding affinity caused by protein amino acid mutations. The in silico experiments show the potential of DDAffinity to be further served as a broad range of applications in protein structure representation learning, as well as in performing a systematic evaluation of protein multiple point mutations effects.

In our future work, we will continue to investigate novel methods to improve and extend the generalization ability and performance of our proposed model. First, we can explore to integrate residue embeddings from protein sequence pre-training language models and/or structure pre-training models. Second, a more efficient weighting function for residue burial needs to be fully explored compared to the present study. Third, since we only consider neighborhood message passing and aggregation of the mutation site in this work, we believe more context information from the protein complex is beneficial. We expect DDAffinity to be highly valuable in the exploration of how multiple point mutations affect binding affinity, protein function, and the role of mutations in diseases.

## Supplementary Material

btae232_Supplementary_Data

## Data Availability

All datasets utilized in this study are publicly available, and the data and source code are available in Github, at https://github.com/ak422/DDAffinity.
