# Supplementary material for: DDAffinity: predicting the changes in binding affinity of multiple point mutations using protein 3D structure
Source: Bioinformatics. 2024 Jun 28;40(Suppl 1):i418–27. doi: 10.1093/bioinformatics/btae232 (PMC11211828; doi:10.1093/bioinformatics/btae232)
Supplement: btae232_Supplementary_Data [file btae232_supplementary_data.zip › btae232_Supplementary_Data/Wang.235.sup.1.pdf]

Supplementary Information for

"DDAffinity: Predicting the changes in binding affinity of multiple point mutations using protein three-dimensional structure"

Guanglei Yu[1,2,3,†,], Qichang Zhao[1,2,†], Xuehua Bi[1,2,3], Jianxin Wang[1,2,*]

[1]School of Computer Science and Engineering, Central South University, Changsha, 410083, China

[2]Hunan Provincial Key Lab on Bioinformatics, Central South University, Changsha, 410083, China

[3]Medical Engineering and Technology College, Xinjiang Medical University, Urumqi 830017, China

*To whom correspondence should be addressed:

Jianxin Wang. Email: jxwang@mail.csu.edu.cn

†The authors wish it to be known that, in their opinion, the first two authors should be regarded as Joint First Authors.

Supplementary Note S1: Performance evaluation on independent test set

Here, we propose a dataset partitioning scheme to minimize overlapping proteins between the testing and training sets. Firstly, we randomly extract proteins from the benchmark dataset without replacement. Then, we add the complexes containing these selected proteins to the training set. We repeat this process until the number of complexes in the training set exceeds 280 (approximately the number of samples in a 5-fold cross-validation). Finally, we assign the remaining complexes to the testing set to ensure that the proteins composing the training and testing sets are distinct. The above scheme is executed 5 times to reduce the randomness in the experiments. The constructed datasets are provided on GitHub (https://github.com/ak422/DDAffinity). We compare DDAffinity with the energy-function method FoldX on these five test sets. The reason for not comparing with DiffAffinity and RDE-Network is that we could not confirm the overlapping protein folds of the independent test sets with their pre-training dataset. The average result on five test sets is shown in Table S1 in Supplementary Material. The result shows that our model outperforms FoldX under the condition of reduced protein fold overlap.

Table S1. Evaluation on the independent test set of SKEMPI2 dataset.

Method	Mutations	r ↑	ρ ↑	RMSE ↓	MAE ↓	AUROC ↑
FoldX	overall	0.309	0.402	1.889	1.343	0.672
	single	0.286	0.348	1.593	1.132	0.653
	multiple	0.280	0.391	2.402	1.834	0.714
DDAffinity	overall	**0.459**	**0.410**	**1.744**	**1.274**	**0.678**
	single	**0.416**	**0.359**	**1.506**	**1.079**	**0.662**
	multiple	**0.481**	**0.444**	**2.168**	**1.717**	**0.724**

Supplementary Note S2: Performance Comparison of DDAffinity and ProteinMPNN

Thanks for the reviewer's suggestion. While ProteinMPNN is not designed to predict ΔΔG, the score it reports could be used as a favorable/unfavorable mutation predictor. By taking wild-type structure and mutant structure as input, ProteinMPNN can get the scores of wild-type structure and mutant structure, respectively, according to the Github: https://github.com/dauparas/ProteinMPNN. Then we can have the change of ΔΔG by subtracting the wild-type score from the mutant score obtained from ProteinMPNN. The comparison results on the SKEMPI2 dataset are shown in Table S2. The results show that DDAffinity gives better predictions than ProteinMPNN.

Table S2. Comparison of DDAffinity and ProteinMPNN on the SKEMPI2 dataset.

**Method**	**Mutations**	r ↑	ρ ↑	**RMSE ↓**	**MAE ↓**	**AUROC ↑**
ProteinMPNN	overall	0.144	0.145	1.57	1.13	0.526
	single	0.171	0.164	1.297	0.929	0.536
	multiple	0.099	0.098	2.282	1.837	0.555
DDAffinity	overall	0.649	0.555	1.208	0.896	0.748
	single	0.617	0.482	1.036	0.772	0.708
	multiple	0.683	0.686	1.674	1.333	0.864

Supplementary Note S3: Exploration of the effect of $\boldsymbol{k}$

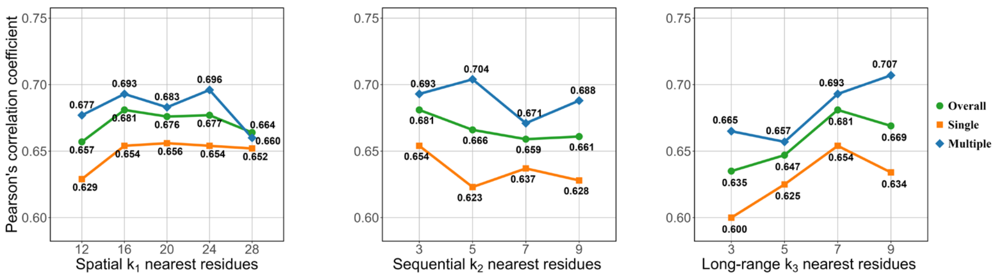

(a) (b) (c)

Figures S1. Illustration of predicted Pearson's correlation coefficient as a function of the number of spatial $\boldsymbol{k_1}$-nearest residues, sequential $\boldsymbol{k_2}$-nearest residues and long-range $\boldsymbol{k_3}$-nearest residues for the overall SKEMPI2, the single point mutations subset of SKEMPI2 and the multiple-point mutations subset of SKEMPI2.

Supplementary Note S4: Visualization on SKEMPI2 dataset

To visualize the predictive ability of DDAffinity, we used scatterplot and histogram to demonstrate the statistical properties of the results derived from DDAffinity, as shown in Figures S2. It is obvious that DDAffinity accurately fits the statistical distribution of multiple point mutation subset of SKEMPI2, as well as the overall SKEMPI2 dataset and single point mutation subset of SKEMPI2.

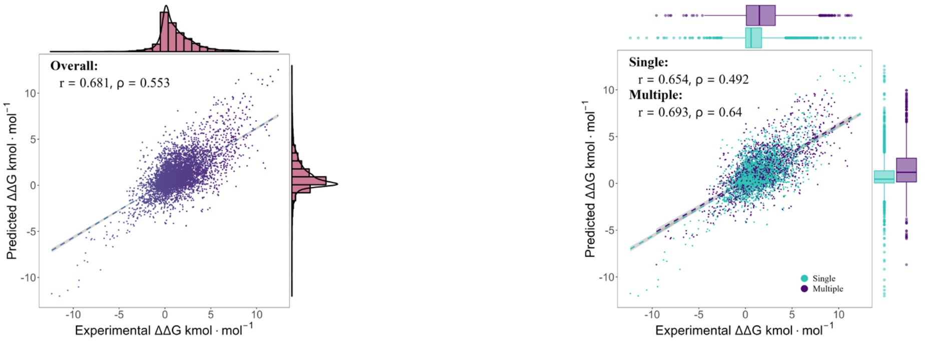

(a) (b)

Figures S2. Visualization of prediction results on SKEMPI2. (a) Pearson's correlation coefficient between experimental $\Delta\Delta Gs$ and predictions of DDAffinity on the overall SKEMPI2 dataset. (b) Pearson's correlation coefficient on the single and multiple point mutation subsets of SKEMPI2.

Supplementary Note S5: Anti-symmetry validation on Ssym dataset

To estimate the anti-symmetric property, we use the Ssym dataset presented in [1], in which the proportion of direct and inverse variations is unbiased. The experimental results are listed in Table S3 below. The Pearson correlation coefficient $\boldsymbol{r}$ between the direct and the corresponding inverse variations is -0.99, and the average bias $<\boldsymbol{\delta}>$ is 0.06. This indicates that DDAffinity shows a perfect anti-symmetrical property.

Table S3 Anti-symmetry performances of DDAffinity on the Ssym dataset [1]

**Method**	**Anti-symmetry**	
	$\boldsymbol{r}$	$<\boldsymbol{\delta}>$
DDGun[a]	-0.99	-0.007
PopMusicSym[a]	-0.77	0.03
SDM[a]	-0.75	-0.32
Maestro[a]	-0.34	-0.58
FoldX[a]	-0.38	-0.47
DDAffinity	-0.99	0.06

[a]These results are taken from [2].

Supplementary References

[1] Pucci, F. et al. (2018). Quantification of biases in predictions of protein stability changes upon mutations. Bioinformatics, 34(21), 3659–3665.

[2] Montanucci, L. et al. (2019). Ddgun: an untrained method for the prediction of protein stability changes upon single and multiple point variations. BMC bioinformatics, 20, 1–10.
